# St. Luke’s Hospital

**Published:** 1871-06

**Authors:** Jno. E. Owen


Article V.—*St. Luke's Hospital. Typhoid Fever, treated with Strychnia. Under care of Jno. E. Owen, M.D.* Reported by C. H. Guibor, M.D., Resident Physician.

Mary A. Smith, æt. 18; born in West Indies; admitted October 2, 1868. The commencement of her illness dates about one week previous to her admission. It began with loss of appetite, soreness, fatigue, and a dull pain in the right side. She was listless, without animation, and all exertion was wearisome. A rigor or chill appeared, with which she went to bed. November 25th. The rigor was followed as usual with fever, pain in the back and head. There was more or less diarrhœa. This was the history gathered from her concerning her condition previous to admission.

On admission:—The cheeks were flushed, skin moist, and tongue coated with a yellowish fur, pain in back and head, tenderness upon pressure in right illiac region, abdomen somewhat swollen, pulse 102, respiration 30, slight cough, with increased bronchial secretion, slight dullness over posterior part of right lung. Auscultation elicited mucous rales, sordes here and there along margin of gums.

Treatment:—Milk every two hours, and if patient asked for a drink, cold milk was given instead of water. In addition to this she took a liberal quantity of nourishing soups, and a tablespoonful three times a day of the following mixture:

℞—Acid Sulph. arom. ʒiii, Strychnia Sulph. gr.$\frac{1}{73}$, Syrup Simpli. ℥v. M.

October 3. Intellect dull, the mind wandering as evening approaches, mutters at night. Pulv. Doverii, gr. x was administered, with tablespoonful doses of wine for three or four hours, on account of great prostration. Tongue dry and brown, but red at tip and edges. Pulse 106.

October 4. Rested well; axilla 100½°, pulse 120, tongue moist; 6 P. M., axilla 101°, pulse 126, delirious; 9 P. M., pulse 132—first fecal discharge (natural) since admission; some wine during the night.

October 5. Rested well after 1 o'clock; 8 A. M., axilla 99°, pulse 114; bowels moved; raw oysters added to the other diet.

October 6. Axilla 98°, pulse 120.

October 7. Rested well; tongue still moist, and cleaning off;

11 A. M., axilla 101°, pulse 120; evening, axilla 100°, pulse 102; natural stool.

October 8. Rested well during the night; noon, axilla 100°, pulse 102; evening, axilla 100°, pulse 120. The patient had a copious, dark, greenish and offensive discharge, resembling blood, from the bowels, quickly followed by two others, with nearly as much pure blood. The Resident gave Dover's Powder and Acetate of Lead. The hemorrhage was checked until 5 A. M. (9th), when it returned.

October 9. Axilla (at 9 A. M.) 99°, pulse 120; recommencement of hemorrhage at 2 P. M.; turpentine emulsion and opium were given; 8 P. M., axilla 100°, pulse 114. (About the end of the second week.)

October 10. Axilla 104°, pulse 120, tongue almost entirely clean.

October 11. Evening, axilla 100°, pulse 108.

October 12. Evening, axilla 100°, pulse 108.

October 13. A. M., axilla 98°, pulse 108; evening, axilla 98°, pulse 108.

October 14. Evening, axilla 98°, pulse 108.

October 15. Evening, axilla 98°, pulse 100. (End of third week.)

October 19. Patient is now convalescing, and is able to be propped up in bed, and even to sit up whilst her bed was being made. Abdomen was more or less tympanitic during last ten days. The rose-colored spots were absent.

Silas Heyes, æt. 28, native of England, was admitted October 5, with the usual symptoms of typhoid fever. He was placed upon liquid food, viz., soups, good milk, and in addition to this the acid and strychnia mixture—tablespoonful three times daily.

October 6. Morning, axilla 98½°, pulse 96; evening, axilla 102°, pulse 112. (Somewhat delirious during the night.)

October 7. Morning, axilla 102°, pulse 108; evening, axilla 102°, pulse 102. Pil. Cath. Co., No. 2, during afternoon. Rose-colored rash; tympanitic; tenderness in right iliac region; bowels have not moved in five days. (End of first week.)

October 8. Bowels moved during the night; A. M., axilla 101½°, pulse 102; evening, axilla 102°, pulse 102.

October 9. Rested well; A. M., axilla 99½°, pulse 102; evening, axilla 102°, pulse 102.

October 10. A. M., axilla 100°, pulse 96 (good night); night, axilla 101°, pulse 102.

October 11. A. M., axilla 100°, pulse 96 (rested well); evening, axilla 102°, pulse 102.

October 12. A. M., axilla 99½°, pulse 100; evening, axilla 100°, pulse 96.

October 13. A. M., axilla 100°, pulse 102; evening, axilla 101½°, pulse 102.

October 14. A. M., axilla 99½°, pulse 96. (End of second week.)

October 15. P. M., axilla 101°, pulse 96.

October 16. A. M., axilla 97°, pulse 96. Bowels not moved for seven days. Castor oil, ℥ss, which operated in a short time. Evening, axilla 98°, pulse 96.

October 17. A. M., axilla 98°, pulse 98; evening, axilla 99°, pulse 90.

October 18. A. M., axilla 98°, pulse 84; evening, axilla 97°, pulse 96.

October 19. Axilla 98°, pulse 84.

October 20. Axilla 97°, pulse 78. (End of third week.)

During the first ten days, the Resident, thinking that the strychnia mixture caused some muscular twitching, discontinued it for twenty-four or thirty-six hours. The tongue became brown, dry and cracked. Upon the renewal of the medicine, the tongue became again moist.

[During the last four years the above has been the treatment of typhoid fever, both in the hospital and in my private practice. There is one noticeable feature in cases treated by strychnia, viz., the dry, brown tongue soon becomes moist, and remains so during the treatment. In several cases, in order in some degree to determine the influence of the mixture, it was discontinued from twenty-four to forty-eight hours. The tongue soon became dry and brownish, but moist again upon its re-administration. In only one case of those treated was the strychnia used alone. The result was the same as when the mixture was used. We have had no opportunity of determining the influence of the mineral acid uncombined with strychnia. Possibly the mineral acids, so long and advantageously employed in the treatment of continued fevers, might have some influence, but we are inclined to believe that the change in the appearance of the tongue is effected mainly through the agency of strychnia, by increasing the nutritive and assimilative functions of the system. Only one case was complicated with intestinal hemorrhage, and this is the only case in which turpentine was used. It was employed in this one only to combat the hemorrhage. In relation to pulse and temperature, the two cases reported are for the most part typical of the others. Of course the employment of any remedy in the later stages of typhoid fever must more or less disappoint us. J. E. O.]

